# Effect of oven and sun drying on the chemical properties, lipid profile of soursop (*Annona muricata*) seed oil, and the functional properties of the defatted flour

**DOI:** 10.1002/fsn3.2380

**Published:** 2021-06-14

**Authors:** Bernard Tiencheu, Agbor Claudia Egbe, Aduni Ufuan Achidi, Eurydice Flore Tiepma Ngongang, Noel Tenyang, Fabrice Tonfack Djikeng, Bertrand Tatsinkou Fossi

**Affiliations:** ^1^ Department of Biochemistry and Molecular Biology Faculty of Science University of Buea Buea Cameroon; ^2^ Department of Biological science Faculty of Science University of Maroua Maroua Cameroon; ^3^ School of Agriculture and Natural Resources Catholic University Institute of Buea Buea Cameroon; ^4^ Department of Microbiology and parasitology Faculty of Science University of Buea Buea Cameroon

**Keywords:** *Annona muricata*, fatty acids, functional properties, lipid profile, oil, Soursop

## Abstract

Soursop seeds present a potential source of edible oil production. This work was aimed at determining the effect of oven and sun drying on the chemical properties and lipid profile of soursop seed oil as well as the functional properties of the defatted seed flour. The chemical properties, lipid profiles, and functional properties of soursop seeds dried for 0, 6, 12, 18, 24, and 30 hr, and 0, 1, 3, and 5 days, respectively, in the oven and on the sun using time T0 as the control sample were determined using oil quality indices, gas phase chromatography, and functionality tests for flours, respectively, with a view of highlighting the potentials of the defatted seed. The result of the study revealed that the chemical properties of oils for oven‐dried and sun‐dried seeds changed with drying technique and time, with iodine value being the more affected parameter, and peroxide value (PV) being the least. The control exhibited the highest free fatty acids (FFAs), peroxide value (PV), thiobarbituric acid (TBA) value, and saponification value compared with dried samples. The fatty acid profiling showed that the predominant fatty acids were C18:1n‐9, C18:2n‐6, and C16:0 and that unsaturated fatty acids (UFA), monounsaturated fatty acids (MUFA), polyunsaturated fatty acids (PUFA), and omega‐fats were not significantly affected by the oven drying time. The mean PUFA content ranged from 31.72% with sundried seeds to 30.92% after 30 hr of oven‐drying and was not significantly affected by the drying technique. The oils contained more n‐6 (30.60%) fatty acids than n‐3 (1.12%). The PUFA/SFA ratios [1.14–1.37] as well as the Atherogenic index (AI) [0.25–0.27] were acceptable because of the recommended range of FAO/WHO. PUFA/SFA, n‐6/n‐3, and Atherogenic index (AI) did not change much with the sun‐drying technique compared with oven‐drying. Flours from sun‐dried seeds had better functional properties than oven‐dried and more than 3 different types of proteins (based on isoelectric points of proteins). It can be concluded that soursop seed contains good quality oil, which can be exploited to improve nutrition. Manufacturers of animal feeds should explore the agro‐industrial use of its oil and defatted seed flour.

## Graphical abstract

Soursop seed presents a potential source for the production of edible oil. This work aimed at determining the effect of oven and sun‐drying on the chemical properties and lipid profile of soursop seed oil as well as the functional properties of the defatted seed flour. The chemical properties, lipid profile, and functional properties of soursop seed were determined using oil quality indices, gas phase chromatography, and functionalities tests for flour, respectively, with a view of highlighting the potentials of the seed. Soursop seed contains good quality oil, which can be exploited to improve nutrition. Manufacturers of animal feeds should explore the use of it oil and defatted seed flour.

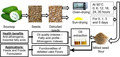

## 1 INTRODUCTION

The origin of most of the *Annona* species is South America and the Antilles; however, wild soursop is thought to have originated from Africa. Four *Annona* species known as bearers of edible fruits are custard apple (*A. reticulata*), sugar apple (*A. squamosa*), cherimoya (*A*. *cherimola*), and soursop (*A. muricata*), which originate from South America (Morton *et al*., 1987, Encina et al., 2014). The wild soursop, *A. senegalensis,* has no precise information on its origin and diversity.

Soursop tree is a small straggly fruit tree growing up to 8 meters high, and it originated from tropical America and West Indies (Acevedo‐Rodriguez & Strong, 2012; Dupriez & De Leener, 1989). However, it is now widely grown in the tropics of both hemispheres (Pamplona, 2004). It is grown in a wide range of soils with good drainage and elevations of up to 1,000 meters and requires a warm humid climate (Kuhnlein et al., 2009).

In many countries, after consumption and exploitation of the fruit for its juice, the seed is discarded and these soursop seeds, like other seeds, contain oil, which can be extracted and used in cooking or as a non‐food such as lubricant, paint, cosmetics, biodiesel, and industrial processes (Bachmann, 2001; Chia‐Hung et al., 2018), and the defatted seed cake may find applications to ensure food security.

It is well known that the most important factors influencing seed longevity are temperature, seed moisture content, and relative humidity (Dickie et al., 1990; Ellis & Roberts, 1980). It is also recognized that the extent to which the potential longevity of a seed is maximized depends on the storage condition as well as on its initial quality (Kameswara et al., 2016; Roberts, 1992). Seeds are generally harvested at high moisture content and need to be dried before storage, and to do this, the attention should be paid to the rate and extent of artificial post‐harvest drying. If drying is too slow, there is a possibility of a reduction in seed quality during the drying process due to seed aging. On the other hand, if seeds are dried rapidly, a large proportion may be lost due to desiccation damage (Ellis & Roberts, 1980; Pammenter & Berjak, 2014). No fixed rule applies to all species. Delay in drying or slow drying together with high temperature (above 25℃) will tend to reduce viability considerably in orthodox seeds. The recommended sophisticated drying methods for safely drying seeds may not be easily implemented in many developing countries due to the high cost of establishing, running, and maintaining such facilities and electricity bills. Therefore, there is a need for low‐cost drying methods to be used as alternatives to such expensive seed drying equipment (Ellis and Roberts, 1985). Traditional sun‐drying and simple oven‐drying are always recommended as commonly used among the variety of methods for seed drying.

Soursop seed could be an oil crop since the seeds and the fruits are economically important in many countries. *Annona muricata* fruits are rich in nutrients, including fat, protein, sugar, fatty acids, and free amino acids, which are indispensable to human life. Many seeds have gained importance due to their potential in lowering cholesterol, delaying human aging, and preventing cancer (Mora‐Escobedo et al., 2015). However, seeds are susceptible to lipid autoxidation because of their high fat and unsaturated fatty acid content. Lipid oxidation occurs during storage, and the formation of oxidation products is associated with changes in the flavor and nutrient value of seed (Jensen et al., 2005). In addition to their nature, the other factors that influence the oxidation of seeds include humidity, temperature, oxygen, and light drying technique (Issaoui & Delgado, 2019; Mexis et al., 2009).

To maintain the quality of seed during storage and hence reduce economic losses, many researchers have studied their lipid oxidation and oxidative stability under different storage conditions. Temperature is one of the most important factors that affect lipid oxidation of seed (Nepote et al., 2006a, 2006b). Processing leads to final products that differ on physicochemical proprieties and nutritional values; hence, grading oils accordingly seem to be primordial and will give consumer more freedom of choice in selecting their wanted sources (Issaoui & Delgado, 2019).

After the extraction of oils from seeds, the defatted flours that result are always good sources of proteins. Flours or proteins possess some functionalities required in food and food formulation. Functional property is defined as: “those physical and chemical properties of proteins that influence their behavior in food systems during preparation, processing, storage, and consumption, and contribute to the quality and organoleptic attributes of food systems.” Some functional properties include bulk density, protein solubility, water and oil absorption capacity, emulsifying properties, and rheological properties (Hefnawy et al., 2012). Many food products owe their functions to food proteins. It is therefore important to understand protein functionality to develop new products and improve existing ones (Genovese & Lajolo, 2001; Hefnawy et al., 2012). Removal of oil from seeds and extraction of juice from pulp generates bi‐products, which may be useful for example in animal feed, and their flours can be successfully incorporated into products (Hefnawy et al., 2012). The functional properties of such bi‐products will further enhance the usage of soursop seeds.

Cameroon is rich in a variety of fruits, but the availability of these fruits is short‐lived due to seasonality and the perishable nature and therefore as reported by Vodouhe et al. (2008) and Probert (2003) seed require drying technique for long storage or as pre‐processing technique. It is therefore important to study one of such seeds to know its potentials for its nutrient content and the quality of its oil. One such fruit seed is *Annona muricata* (soursop). It is postulated that this underutilized and unexploited fruit seed may be of great potential in combating micronutrients deficiency or used as a feed substitute (Degnon et al., 2013; Dupriez & De‐Leener, 1989). However, very little work has been done with soursop in Cameroon to highlight the potentials of its seeds; thus, this work is aimed at evaluating the effect of sun and oven‐drying on the chemical properties and lipid profile of soursop seeds as well as functional properties of the defatted seed flour.

## 2 MATERIALS AND METHODS

### 2.1 Sample collection

Fifty mature soursop fruits from the same source were purchased from vendors at Muea market, South West Region of Cameroon. The fruit's maturity was determined by its dark green skin with smooth, numerous fleshy spines. The samples were transported to the Life Science Laboratory, University of Buea, for sample preparations and analyses.

### 2.2 Sample preparation and drying

#### 2.2.1 Sample preparation

**FIGURE 1:**
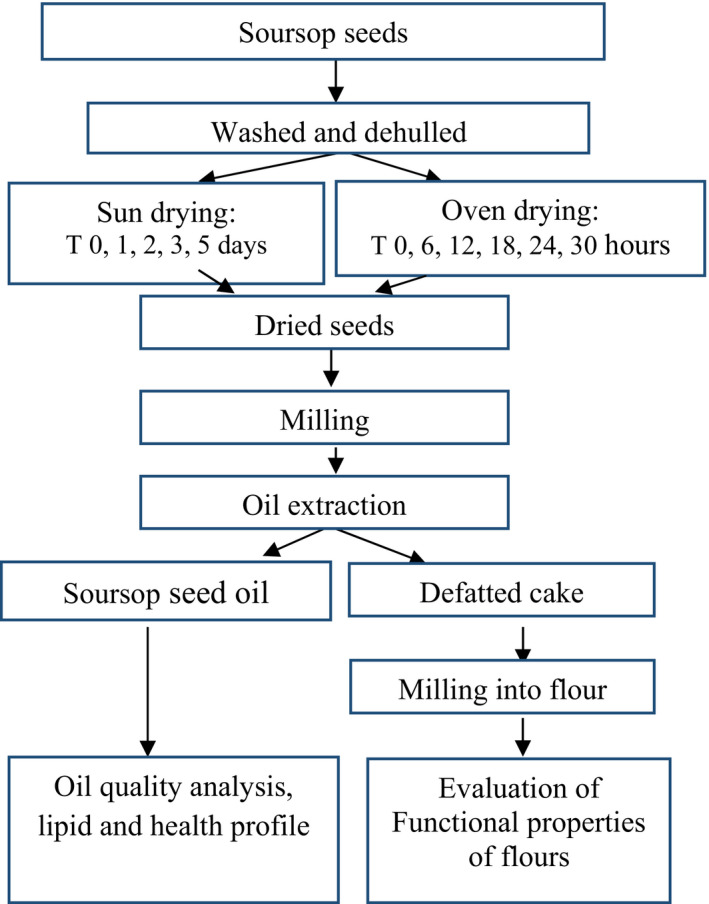
A flow diagram for seed drying, oil extraction, oil quality indices, and functional properties of defatted seed flour

Seeds were removed manually by hands from each ripe soft fruits (average number of seeds per fruit calculated were approximately 80), were washed, manually dehulled, and dried by two drying methods (oven and sun drying) (Figure 1). The dried seeds were milled. Oil was extracted from the flour and analyzed for oil quality indices, while the defatted flour was evaluated for functional properties. A flow diagram of the procedure for analyses of the seed is shown in Figure 1. A portion of the seeds was milled without drying using a Blending machine and served as the control (raw un‐dried sample: To).

#### 2.2.2 Oven Drying

The oven‐drying of the dehulled seed samples was done using an electric conventional oven drier at a constant temperature of 50℃ for 0, 6, 12, 18, 24, and 30 hr for each sample. After reaching the drying time, 300g of each sample was then ground with an electrical blender. Each dried refined flour was immediately soaked in the solvent for the oil extraction.

#### 2.2.3 Sun drying

Sun drying was carried out by placing the fresh seeds on a wire gauze and putting them directly in the hot, humid subtropical sun. The sun drying was conducted on 4 samples for 0, 1, 3, and 5 days, between 9 a.m. and 3 p.m. daily. The average daily temperature during this period was 33℃, and relative humidity was 67%. After reaching the drying time, 300g of each dried sample was milled to yield fine flour using an electric blender and was immediately soaked in the solvent for the extraction oil.

### 2.3 Oil extraction and analysis

#### 2.3.1 Oil extraction or defatting of seeds

**FIGURE 2:**
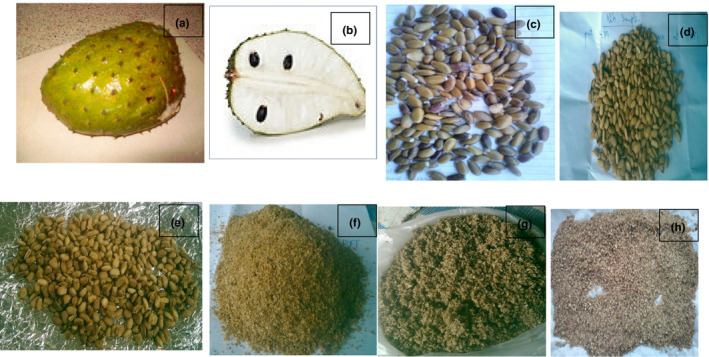
Soursop fruit (a), pulp (b), fresh seeds (c), dry seeds (d, e), oven‐dried seed flour (f), fresh seed flour (g), and sundried seed flour (h)

The extractions were performed according to the method described by Womeni et al. (2002). 100 g flour of each sun‐dried or oven‐dried sample was mixed with 500 ml of hexane, macerated, and stirred many times within 24 hr. Upon maceration, the homogenate was filtered and concentrated in a vacuum using rotary vapor (40℃). The resulting extract (oil) and the defatted cake (Figure 2) were placed in an oven at 35℃ for 2 days to remove residual solvent, weighed, and stored for two days at −20℃ in dark containers for further use.

#### 2.3.2 Determination of oil quality indexes

Acid value (expressed as % free fatty acids (% FFA)), iodine (IV), peroxide value (PV), saponification value (SV), and thiobarbituric acid value (TBA‐V) were assessed on oils extracted on the previous oven‐dried and sun‐dried samples as recommended by standard NFT60‐204 of the French Association for Standardization (AFNOR, 1981).

#### 2.3.3 Fatty acid composition

The fatty acid composition of the oil was investigated after conversion of the fatty acid methyl esters (FAME) by using boron trifluoride‐methanol method. The lipids were saponified and esterified for fatty acid analysis by the method of Metcalfe et al. (1966). The fatty acid methyl esters (FAMEs) were analyzed on a Hewlett‐Packard (HP) 5,880 Gas Chromatograph (GC) with a Flame Ionization Detector (FID). The esters were separated on a 50 m × 0.20 mm I.D. wall‐coated open tubular fused silica capillary column coated with Carbowax 20 M. Column injector and detector temperatures were 200 and 300℃, respectively. The carrier gas was helium with a flow rate of 1.5 ml/min and the split ratio 100:1, and the working pressure was 33MPa. Identification was achieved by comparison to retention times of authentic standards, and their concentrations determined based on the area occupied by each peak. The fatty acids types (UFA, SFA, MUFA, PUFA, n‐3, and n‐6) were done by addition. The atherogenic indexes were calculated according to Ulbricht and Southgate (1991): Atherogenic index = (C12:0 + 4 × C14:0 + C16:0) / [∑ MUF A + ∑ (n ‐ 6) + ∑ (n ‐ 3)].

### 2.4 Determination of functional properties of defatted seed flour

Functional properties were conducted on residual defatted oven drying and sun‐drying flour. Water absorption capacity (WAC) and oil absorption capacity (OAC) were determined using the method described by Chau et al. (1997). Bulk density (BD) (the loose bulk density and packed bulk densities) and emulsion activity (EA) were determined by the method described by Okezie and Bello (1988). For Swelling Index (SI), the method of Abbey and Ibeh (1998) was employed. The dispersibility of the blends was determined as described by Kulkarni et al. (1991).

#### 2.4.1 Protein solubility

Protein solubility was tested in the pH ranges of 1 to 10. One gram of each defatted flour was suspended in 5ml distilled water, and the pH of the suspensions was adjusted to a specific value using 0.1N HCl or NaOH solutions. These suspensions were agitated over a shaker for one hour at room temperature, and the pH was checked and re‐adjusted in the range of 1.0 to 10 and then centrifuged at 4000xg for 15 min. The soluble proteins were determined by the method of Biuret at 540nm (Ken‐ichiro Kanaya & Keitaro Hiromi, 1987) using BSA as standard. Protein solubility was expressed as a percentage of the total protein of the original sample that was present in the soluble fraction.

### 2.5 Statistical analysis

All the data obtained were subjected to one‐way analysis of variance (ANOVA), results were expressed as means ± *SD* of triplicate analysis, and the means were separated using new Duncan's multiple range test in Statistical Package for Social Sciences (SPSS) version 17.

## 3 RESULTS AND DISCUSSION

### 3.1 Chemical properties of oil extracted from sun‐dried and oven‐dried seeds

The chemical properties of the oil are among the most important properties that determine the present conditions of the oil. Free fatty acid, thiobarbituric acid value, and peroxide values are valuable measures of oil quality. The iodine value is a measure of the degree of unsaturation of the oil. Results of the chemical properties of raw (0‐hr drying, i.e., control sample), oven‐dried (OVD), and sun‐dried (*SD*) soursop seeds are presented in Table 1. %FFAs were low (*p* ≤.05) in all treated seeds samples compared to the control (untreated sample) particularly with sun drying seeds. The acid values in the oils decreased after drying in the sun and oven compared to the control sample. However, these changes were not significant (*p* >.05) with the drying time. Dehydrations have been reported to have a reduction effect on acid value due to limitation of water for lipase hydrolysis or due to denaturation of the enzyme. The values obtained in this study were higher than the stipulated permitted value of 2.5% FFA for virgin oil (FAO/WHO, 2009). The acid values in this study indicate that the oil samples will not be stored for long and will not have a long shelf life since free fatty acid can easily undergo oxidation than its esterified counterpart. A low FFA content (0.91%) was reported by Adepoju et al. (2014). Kimbonguila et al. (2010) reported a value of FFA 13.5% and 14.2% for soursop seed oil extracted, respectively, by Blye & Dyer and Soxhlet methods. Compared to the control, the PV were significantly lower(*p* ≤.05) than the control and ranged from 1.05 to 9.10 meqO_2_/kg and 2.42 to 9.10 meqO_2_/kg for oven‐dried and sun‐dried seeds, respectively, indicating that these oils were more stable for oxidative deterioration or that the primary products of oxidation had been transformed into secondary products. Despite the decrease compared to the control, all values were within the recommended range for good quality oil as prescribe by FAO/WHO (2009) (10 meq/kg). The combination of high iodine value and low peroxide value suggests that the oil could also be stored for a long period without deterioration. These also demonstrate that the oil possesses the desirable qualities of edible oils. The soursop oil could therefore be used for food purposes and as a feedstock in the industries (Adepoju et al., 2014).

**TABLE 1:** Effect of drying methods of the seed on the chemical properties of the oil extracted

	% FFA	PV (meq O_2_/kg)	IV (g I_2_/100g)	TBA‐V (mg MDA/g)	SV (mgKOH/g)
Hours Oven‐drying					
0	8.39_a_ ± 0.90	9.10_a_ ± 1.60	164.00_a_ ± 0.34	0.13_a_ ± 0.12	151.30_a_ ± 1.29
6	5.54_b_ ± 1.40	2.01_b_ ± 0.84	117.6_b_ ± 3.76	0.61_a_ ± 0.10	151.14_a_ ± 1.11
12	4.77_b_ ± 0.35	2.33_b_ ± 0.82	110.22_b_ ± 0.32	0.85_a_ ± 0.36	157.11_a_ ± 0.83
18	6.34_b_ ± 1.32	1.32_b_ ± 0.50	66.5_c_ +1.68	0.65_a_ ± 0.32	167.35_a_ ± 1.84
24	5.08_b_ ± 1.46	1.05_b_ ± 0.13	66.3_c_ ± 1.33	0.86_a_ ± 0.21	155.57_a_ ± 2.06
30	5.28_b_ ± 1.47	1.81_b_ ± 0.73	82.21_c_ ± 0.44	0.89_a_ ± 0.28	153.28_a_ ± 0.53
Days Sun‐drying					
0	8.39_a_ ± 0.90	9.10_a_ ± 1.60	164.00_a_ ± 0.35	0.13_a_ ± 0.12	151.30_a_ ± 1.29
1	2.80_b_ ± 1.02	2.34_b_ ± 34.34	66.7_b_ ± 0.36	2.93_b_ ± 0.41	147.70_a_ ± 0.05
3	7.49_a_ ± 0.70	2.56_b_ ± 2.00	56.63_b_ ± 1.33	1.90_b_ ± 0.08	149.40_a_ ± 0.45
5	5.48_b_ ± 0.74	2.42_b_ ± 0.66	55.63_b_ ± 1.64	5.19_b_ ± 2.63	144.50_b_ ± 0.42

Note

a, b, c: Values with same superscript on the same column are significantly different (*p* <.05).

Abbreviations: FFA, Free fatty acids; IV, Iodine value; PV, Peroxide value; SV, Saponification value; TBA‐V, Thiobarbituric acid value.

The degree of unsaturation of oils and fats is generally measured by the determination of the iodine value. Generally, during lipid oxidation, the double bonds of unsaturated fatty acids are attacked by free radicals, resulting in the reduction of the number of unsaturation (Tynek et al., 2001). Iodine values range between 55.63 and 164 gI_2_/100g. These iodine values significantly decrease with oven drying compared to the control (raw seed oil, no drying). The lowest iodine value was obtained after 18 hr of oven drying and from 3 days of sun drying. These results are in agreement with those reported by Taiwo et al. (2008)

The TBA test is an easy and quick technique widely used in the assessment of the secondary oxidation state of oils and fats. It especially gives an idea of the concentration of malonaldehyde present, which is one of the secondary oxidation products of oils and fats (Iqbal & Bhanger, 2007). Table 1 revealed that a part of the control recorded a TBA value of 3.13; the secondary product of oxidation obtained from sun‐dried seeds had a higher concentration (*p* ≤.05) as compared to those obtained from oven‐dried seeds. TBA‐V of oils was not significantly affected oven‐dried seeds. Five days of oven‐dried seeds gave oil having the highest TBA value or products of oxidation (5.19) than those dried in an oven. This is in accordance with the report of Womeni et al. (2010) who obtained the same conclusion during the drying process of palm kernel, since sun (UV light) is recognized as a powerful initiator of lipid degradation. It has been demonstrated that sunlight facilitates the initiation of lipid oxidation, which leads to the formation of free radicals, which can easily react with molecular oxygen and form hydroperoxides at the propagation stage and aldehyde at the termination phase (Lobo et al., 2010), so the high PV and TBA‐V of traditionally sun‐dried seeds indicates its poor resistance toward peroxidation during storage. These results are in line with those reported by Tenyang et al. (2017) who showed that the peroxide values of oils extracted from two varieties of sesame seeds was increased with drying temperature and time.

SV gives an idea of the fatty acid molecular weight or length. In this study, the saponification values of the oils were not significantly affected by oven or sun drying of seeds, except day 5 where the TBA value was significantly high (*p* ≤.05) compared with the control. SV ranged between 144 and 167 mg KOH/g oil. These values were lower than 235.46 proposed by Adepoju et al. (2014) on the same soursop seeds.

### 3.2 Fatty acid composition of oil extracted from oven‐dried seeds

In the present study, table 2 revealed that UFA, MUFA, PUFA, and omega‐fats were not significantly affected by the oven drying time. This is the TBA value that was also not affected, indicating nonoxidation or low oxidation of fatty acid to secondary products of oxidation since unsaturated fatty acids are more susceptible to disappearances, changes, and oxidations. The predominant fatty acids were C18:1c‐9, C18:2 n‐6, and C16:0. The high levels of C18:1 c‐9 in the current study are desirable since MUFA (44.43%) has neutral effect on human cholesterol levels. Oleic acid increases the concentration of HDL cholesterol and lowers the concentration of LDL. Essential PUFA (linoleic and α‐linolenic acid) is beneficial to human health due to their antiatherogenic, antithrombotic, and anti‐inflammatory effects (Panagiotis et al., 2019). However, n‐3 is preferable over high levels of n‐6 (Panagiotis et al., 2019). After the drying technique, the oils were at least 23% saturated and the remaining 77% made of unsaturated fatty acids, among which MUFA represented 44% and PUFA 31%. The mean content of PUFA ranged from 31.72% in undried seeds to 30.92% after 30 hr of oven‐drying and was not significantly affected by the drying duration. The oils contained more n‐6 (30.60%) fatty acids than n‐3 (1.12%).

**TABLE 2:** Effect of oven‐dry on Fatty acid profile and health lipid indices of soursop seed oil

% Fatty acid	Common name	Oven drying duration (hours)					
		0	6	12	18	24	30
C16:0	Palmitic acid	19.4	19.45	19.42	19.30	19.5	19
C16:1 Δ^9^	Palmitoleic acid	2.03	2.08	2.05	1.93	2.13	1.63
C18:0	Stearic acid	4.23	4.28	4.25	4.13	4.33	3.83
C18:1Δ^9^	Oleic acid	42.4	42.45	42.42	42.30	42.5	42
C18:2 Δ^9.12^ (*n*−6)	Linoleic acid	30.6	30.65	30.62	30.50	30.7	30.2
C18:3 Δ^9.12.15^ (*n*−3)	α‐Linolenic acid	1.12	1.17	1.14	1.02	1.22	0.72
C20:0	Arachidic acid	0.22	0.27	0.24	0.12	0.32	0.18
⅀SFA	Total saturated fatty acids	23.85	24	23.91	23.55	24.15	22.65
⅀UFA	Total unsaturated fatty acids	76.15	76	76.09	76.45	75.85	77.35
⅀MUFA	Total monounsaturated fatty acids	44.43	44.53	44.47	44.23	44.63	43.63
⅀PUFA	Total polyunsaturated fatty acids	31.72	31.82	31.76	31.52	31.92	30.92
⅀(*n*−6)	Total omega−6	30.60	30.65	30.62	30.5	30.7	30.2
⅀(*n*−3)	Total omega−3	1.12	1.17	1.14	1.02	1.22	0.72
PUFA/SFA	PUFA/SFA Ratio	1.33	1.33	1.33	1.34	1.32	1.37
*n*−6/*n*−3	Omega−6/ omega−3 ratio	27.32	26.20	26.86	29.90	25.16	41.94
AI	Atherogenic index	0.25	0.26	0.26	0.25	0.26	0.25

Note

∑SFA: Total saturated fatty acids; ∑MUFA: Total monounsaturated fatty acids; ∑PUFA: Total polyunsaturated fatty acids; ∑UFA: Total unsaturated fatty acid (MUFA +PUFA); ∑*n*‐6 PUFA: Total *n*‐6 PUFA fatty acids; ∑*n*‐3 PUFA: Total *n*‐3 PUFA fatty acids; AI: Atherogenic index; Fatty acids are expressed in term of % (in 100g of oil). IA = [C12 : 0 + (4 × C14 : 0) + C16 : 0]/SUFA

The PUFA/SFA and *n*‐6/*n*‐3 PUFA ratios, atherogenic index (AI), and thrombogenic index (TI) are commonly used to assess nutritional value and consumer health. In general, a ratio of PUFA to SFA above 0.45 and a ratio of *n*‐6/*n*‐3 below 4.0 are required in the diet to combat various “lifestyle diseases” such as coronary heart disease and cancers (Simopoulos, 2002). The PUFA/SFA ratio was acceptable within the recommended range; if the fat is highly unsaturated like these oils, it is considered to have non‐detrimental effects on human health (Monteiro et al., 2006), but the omega ratios were higher (27%) because soursop seed oil contains much more linoleic acid than linolenic acid. *n*‐6/*n*‐3 ratios in this study were considerably higher than the recommended values. A very high *n*‐6/*n*‐3 ratio was also found by Kimbonguila et al. (2009) studies. The higher *n*‐6/*n*‐3 ratio with soursop oil was attributed also to high concentrations of C18:2*n* 6. However, some authors consider that an index such as PUFA/SFA may not be an adequate way to evaluate the nutritional value of fat because some SFA does not increase plasma cholesterol and ignore the effects of MUFA (Orellana et al., 2009). More recent lipid research would suggest that C12:0 and C14:0 have a greater total cholesterol‐raising effect than C16:0, whereas C18:0 has a neutral effect on the concentration of total serum cholesterol, including no apparent impact on either LDL or HDL (Daley et al., 2010; Mensink & Katan, 1989) our soursop seeds oil lacked C12:0 and C14:0, therefore no hypercholesterolemic increasing effects. Hence, it has been shown that the C12:0, C14:0, and C16:0 FA are associated with an increase in plasmatic cholesterol concentrations when they are present in human diets. This association is stronger for C14:0, which has the potential to increase cholesterol concentrations 4 to 6 times greater than C16:0 (Bressan et al., 2011; Mensink & Katan, 1989).

Stanley et al. (2007) have proposed that it is more important to evaluate the total amount of dietary PUFA than their respective ratio. According to Wijendran and Hayes (2004), epidemiological and clinical studies have established that the *n*‐6 FA, linoleic acid (LA), and the *n*‐3 fatty acids, linolenic acid (LNA), eicosapentaenoic acid (EPA), and docosahexaenoic acid (DHA) collectively protect against coronary heart disease. Linoleic acid is the major dietary fatty acid regulating low‐density lipoprotein cholesterol metabolism by downregulating low‐density lipoprotein cholesterol production and enhancing its clearance. Atherogenic index (AI) takes into account the different effects that single FA might have on human health and in particular on the probability of increasing the incidence of pathogenic phenomena, such as atheroma formation. AI is highest for most atherogenic thrombogenic dietary components. In the present study, AI was favorable and was all from 0.25. It is assumed that AI below 1 is beneficial for human health. Also, AI was very favorable, since such good indices were caused by a low content of SFA, especially palmitic acid, the absence of stearic acids, and a high content of *n*‐6 PUFA and MUFA. The AI values obtained are lower than those reported for reared and wild sharp snout sea bream, *Diploduspuntazzo* Rueda et al. (2001). The IA indicates the relationship between the sum of SFAs and the sum of unsaturated fatty acids (UFAs). The main classes of SFAs, which include C12:0, C14:0, and C16:0, except C18:0, are considered pro‐atherogenic (they favor the adhesion of lipids to cells of the circulatory and immunological systems) (González‐Félix et al. 2016; Monteiro et al., 2018). UFAs are considered to be anti‐atherogenic as they inhibit the accumulation of plaque and reduce the levels of phospholipids, cholesterol, and esterified fatty acids (González‐Félix et al. 2016; Monteiro et al., 2018). Therefore, the consumption of foods or products with a lower IA can reduce the levels of total cholesterol and LDL‐C in human blood plasma (Kholif et al., 2018). The value ranges from 0.084 to 0.55 for crops, 0.21 to 1.41 for fish, and 0.165 to 1.32 for meat, 1.42 to 5.13 for dairy products was obtained by Jiapeng and Hongbing (2020).

### 3.3 Fatty acid composition of oil extracted from sun‐dried seeds

In the present study, table 3 revealed that the UFA, MUFA, PUFA, and omega fats were also not significantly affected by the sun drying time but when compared with the oven‐drying technique, sun‐drying registered MUFA, PUFA, and omega fatty acids less than the ones obtained during oven drying. This is an indication that exposure to sunlight is a powerful catalyst or initiator of lipid oxidation through UV light, as well as their action on the endogenous lipoxygenase enzymes. These same observations were reported during oven and sun‐drying of shea butter kernel, palm kernel seed, insect, and shrimps (Ajifolokun et al., 2019; Tiencheu et al., 2013; Womeni et al., 2002, 2010). Like with oven‐dried seeds, a total of 7 fatty acids were also identified with sun‐dried seeds. The main MUFA fatty acids identified were also oleic acid (42.40%), linoleic acid (30.6%), stearic acid (0.00%), and palmitic acid (19.4%).

**TABLE 3:** Effect of sundry on Fatty acid profile and health lipid indices of soursop seed oil

% Fatty acid	Common name	Duration of sun‐drying (days)			
		0	1	3	5
C16:0	Palmitic acid	19.40	19.31	19.59	20.27
C16:1 Δ^9^	Palmitoleic acid	2.03	1.69	1.75	1.71
C18:0	Stearic acid	4.23	4.17	4.21	5.05
C18:1 Δ^9^	Oleic acid	42.40	42.37	42.25	41.00
C18:2 Δ^9.12^ (*n*−6)	Linoleic acid	30.60	30.61	30.65	29.60
C18: Δ^9.12.15^ (*n*−3)	α‐Linolenic acid	1.12	1.14	0.82	0.76
C20:0	Arachidic acid	0.22	0.62	0.57	1.34
⅀SFA	Total saturated fatty acids	23.85	24.09	24.38	26.66
UFA	Total unsaturated fatty acids	76.15	75.91	75.63	73.34
⅀MUFA	Total monounsaturated fatty acids	44.43	44.06	43.99	44.08
⅀PUFA	Total polyunsaturated fatty acids	31.72	31.74	31.47	30.36
⅀(*n*−6)	Total omega‐ 6	30.60	30.61	30.65	29.60
⅀(*n*−3)	Total omega −3	1.12	1.14	0.82	0.76
PUFA: SFA	PUFA/SFA Ratio	1.33	1.32	1.29	1.14
*n*−6/*n*−3	Omega‐ 6/ omega −3 ratio	27.32	26.84	37.38	38.95
AI	Atherogenic index	0.25	0.25	0.26	0.27

NOTE

∑SFA: Total saturated fatty acids; ∑MUFA: Total monounsaturated fatty acids; ∑PUFA: Total polyunsaturated fatty acids; ∑UFA: Total unsaturated fatty acid (MUFA +PUFA); ∑*n*‐6 PUFA: Total *n*‐6 PUFA fatty acids; ∑*n*‐3 PUFA: Total *n*‐3 PUFA fatty acids. AI: Atherogenic index; Fatty acids are expressed in term of % (in 100g of oil). IA = [C12 : 0 + (4 × C14 : 0) + C16 : 0]/ ∑UFA

The analysis also showed that the oil contains a lesser amount of UFA than in the oven drying process. SFA was not significantly (*p* <.05) affected by sun‐drying. According to European standards, the concentration of linolenic acid should not exceed the limits of 12.0% (Akbar et al., 2009). However, the values obtained here were higher. The oleic acid content was very high. It has been reported that oil rich in oleic acid will possibly improve the fuel property of biodiesel (Knothe, 2005). Comparing its fatty oil composition with other edible oils (Chowdhury et al., 2007; Kowalski, 2007), it is found that the oil could be useful for the production of biodiesel. *Annona* fatty oil can also be used for edible purposes after detoxification as the oil showed a high amount of unsaturated fatty acids.

PUFA/SFA, n‐6/n‐3, and atherogenic did not change much with the sun‐drying technique compared to the oven, except a slight reduction of UFA and PUFA with sun‐drying duration and an increase of AI from 0.25 for the control to 0.27 for the last day of sun‐drying. Despite this increase, the value of AI remained acceptable (lower than 1 as stated by (González‐Félix et al. (2016) and Monteiro et al. (2018).

### 3.4 Functional properties of soursop seed flour

Functional properties describe how ingredients behave during preparation and cooking, how they affect the finished food product in terms of look, taste, and feel.

**FIGURE 3:**
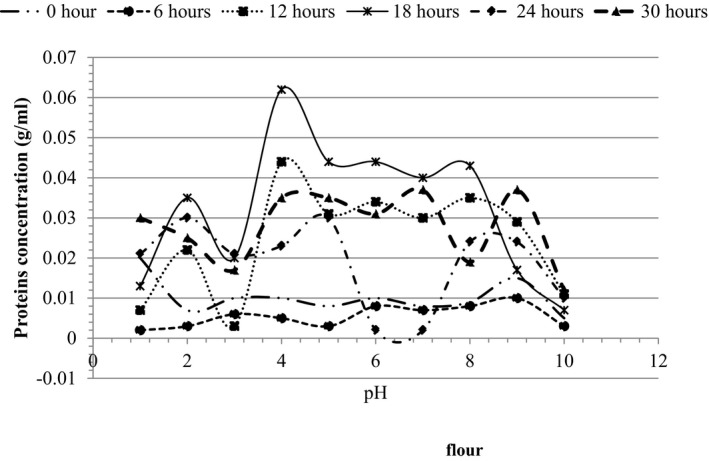
Protein solubility for oven‐dried soursop seed flour

**FIGURE 4:**
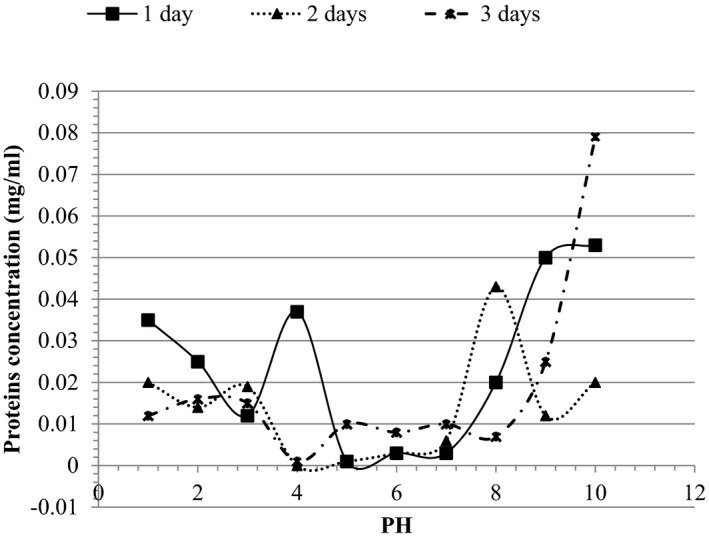
Protein solubility of sun‐dried soursop seed flour

Functional properties of the oven and sun‐dried soursop seed flours are shown in Table 4, while protein solubilities are shown in Figures 3 and 4.

**TABLE 4:** Functional properties of soursop flour from oven‐dried and sundried seed

	OAC (%)	WAC (%)	LBD (g/ml)	PBD (g/ml)	SI(ml)	% Dispersibility	EA (%)
Oven dried							
(0 hr)	250_a_ ± 0.71	310_a_ ± 0.30	0.33_a_ ± 0.04	0.45_a_ ± 0.08	1.03_a_ ± 0.07	70.5_a_ ± 0.21	10.5_a_ ± 0.05
(6 hrs)	275_a_ ± 0.43	275_a_ ± 0.14	0.43_a_ ± 0.4	0.51_a_ ± 0.00	1.01_a_ ± 0.00	64.0_a_ ± 0.10	16.0_b_ ± 0.01
(12 hrs)	335_a_ ± 0.80	300_a_ ± 0.76	0.36_a_ ± 0.6	0.53_a_ ± 0.02	0.99_a_ ± 0.10	66.0_a_ ± 0.01	24.4_c_ ± 0.50
(18 hrs)	320_a_ ± 1.12	285_a_ ± 0.50	0.38_a_ ± 0.0	0.53_a_ ± 0.04	1.01_a_ ± 0.01	67.0_a_ ± 0.05	30.5_d_ ± 1.07
(24 hrs)	260_a_ ± 0.10	325_a_ ± 0.43	0.38_a_ ± 0.9	0.53_a_ ± 0.06	0.99_a_ ± 0.08	64.5_a_ ± 0.10	15.6_e_ ± 0.02
(30 hrs)	305_a_ ± 0.25	285_a_ ± 0.18	0.37_a_ ± 0.1	0.51_a_ ± 0.01	1.02_a_ ± 0.04	65.5_a_ ± 0.20	20.7_f_ ± 0.03
Sun‐dried							
(0day)	250_a_ ± 0.71	310_a_ ± 0.30	0.33_a_ ± 0.04	0.45_a_ ± 0.08	1.03_a_ ± 0.07	70.5_a_ ± 0.21	10.5_a_ ± 0.05
1 day	350_a_ ± 2.0	325_a_ ± 0.45	0.31_a_ ± 0.03	0.48_a_ ± 0.04	1.03_a_ ± 0.04	57.0_a_ ± 0.06	15.0_a_ ± 0.07
3 days	325_a_ ± 1.40	310_a_ ± 0.10	0.32_a_ ± 0.02	0.51_a_ ± 0.08	1.05_a_ ± 0.08	51.5_a_ ± 0.29	14.2_b_ ± 0.09
5 days	300_a_ ± 2.40	350_a_ ± 0.19	0.32_a_ ± 0.04	0.50_a_ ± 0.00	1.03_a_ ± 0.02	58.0_a_ ± 0.31	44.0_c_ ± 0.13

Abbreviations: EA, Emulsion Activity; LBD, Loose Bulk Density; OAC, Oil Absorption Capacity; PBD, Packed Bulk Density; SI, Swelling Index; WAC, Water Absorption Capacity.

#### 3.4.1 Effect of drying on OAC, WAC, LBD, PBD, SI, EA, and Dispersibility of flours

Apart from emulsion activity (EA) all other functional properties were not statistically different (*p* >.05) for both oven and sun‐dried flours with respect to drying times. However, the oven‐dried flours had lower OAC, WAC, swelling power, EA, and protein solubility, while sun‐dried flours had lower bulk density and dispersibility.

OAC is defined as the difference in the flour weight before and after its oil absorption (Giami et al., 1994). It is of great importance since oil acts as a flavor retainer and also increases the soft texture to the mouthfeel of foods, especially bread and other baked foods (Kinsella, 1976). They are also important because of their storage stability and particularly in the rancidity development (Siddiq et al., 2010). It was observed that the oil absorption capacity of sun‐dried samples was higher compared to the oil of oven‐dried samples. However, there was no significant difference at *p* >.05. The values obtained for oil absorption capacity compared favorably with oil absorption capacity reported for potato flour by Jones et al. (2000); but higher than those of flour from Ghanaian breadfruit (150%–250%) (Appiah et al., 2011). Variations in OAC might be partially due to the different proportions of non‐polar side chains of the amino acids on the surfaces of their protein molecules. According to Kinsella (1976), more hydrophobic proteins show superior binding of lipids, indicating that non‐polar amino acid side chains bind the chains of fats.

Water absorption capacity (WAC) indicates the amount of water available for gelatinization (Kulkarni et al., 1990). It is used in determining the suitability of utilizing material in baked foods such as bread where high WAC is needed. It was observed that the range for water absorption capacity of sun‐dried samples was higher, compared to oven‐dried samples; however, there was no significant difference (*p* >.05). The observed WACs of this study were comparable to those of flours from tubers cultivated in Côte d'Ivoire (155.31%–351.14%) (Koné et al., 2014) but lower than those recorded for potato flour (752%), which is not higher than those for wheat flour (140%) by Adeyeye and Aye (1998). The high water absorption capacity observed for sun‐dried flour could be attributed to its higher swelling of fiber and carbohydrate content (Narayana & Narsinga, 1982). Hence, sun‐dried flour would be better in food products like dough and baked products.

Bulk density (BD) is a reflection of the load (heaviness) that the sample can carry if allowed to rest directly on one another. The lower the bulk density, the higher the number of flour particles that stays together and thus increasing the energy content that could be derivable from such diets (Ikpeme‐Emmanuel et al. 2009). It is important for determining packaging requirements, material handling, and application in wet processing in the food industry (Shittu et al., 2007). Loose bulk density represents the lowest attainable energy without compression (Achidi et al., 2016). Oven‐dried samples had a higher LBD (ranging from 0.33 to 0.43g/ml) compared to the sun‐dried samples (ranging from 0.31 to 0.32g/ml); however, they were not statistically different (*p* <.05). Packed bulk density (PBD) represents the highest attainable density with compression (Achidi et al., 2016). PBD of oven‐dried flour was higher compared to the sun‐dried flour but not statistically significant (*p* >.05).

The results for bulk density imply that less packaging material would be required for the sun‐dried flour (since it has a lower bulk density compared to the oven‐dried samples), as bulk density indicates the relative volume of packaging materials required. Padmashree et al. (1987) had observed that higher bulk density is desirable for the greater ease of dispersibility and reduction of paste thickness. The values obtained for sun and oven‐dried flour were found to be higher than potato flour and green gram flour reported by Akpata and Akubor. (1999) in their study of functional properties of legume flours. Thus, the high bulk density of oven‐dried flour and sun‐dried flour indicates that they would serve as good thickeners in food products since they will have good dispersibility.

There was no statistically significant difference (*p* >.05) between the swelling power of the sun and oven‐dried flours. However, the values were much lower than the values reported by Misael and Aguilera (2006) for potato (42.9ml), followed by green gram (19.80ml) and wheat flour (17.60ml). Differences in carbohydrate content, the particle size of flour, methods, or unit operation have been shown to strongly influence swelling power. The finer flours have a greater swelling capacity (Sandhya & Bhattacharya, 1989), and flours with high swelling power are more suitable for dough and baked products.

Dispersibility is a measure of reconstitution of flour or starch in water, the higher the dispersibility, the better the sample reconstitutes in water (Adebowale et al., 2008) and gives fine constituent during mixing (Adebowale et al.,^2008^, ^2012^). Oven‐dried flour had higher values than sun‐dried flour, but there was no significant difference (*p* <.05). The low dispersibility of the sun‐dried flour probably implies that the samples will have a lump formation tendency during preparation. The higher dispersibility value exhibited by oven‐dried starches is indicative of their ability to produce a smooth or consistent paste.

The emulsifying properties are usually attributed to the flexibility of solutes and exposure to hydrophobic domains. Food emulsions are thermodynamically unstable mixtures of immiscible liquids. The formation and stability of the emulsion are very important in food systems such as salad dressing (Fekria et al., 2012). The emulsion activity of sun‐dried flour was significantly (*p* <.05) higher than oven‐dried flour. The higher emulsion of the sun‐dried flour could be a result of differences among the emulsifying properties related to the protein contents (soluble and insoluble) and other components (starch, fat) contents of flour. The capacity of proteins to enhance the formation and stabilization of emulsion is important for many applications, such as in cakes, coffee whiteners, and frozen desserts. Thus, sun‐dried soursop seed flour could be used for these applications.

#### 3.4.2 Effect of pH on protein solubility

Proteins solubilities at different pH are shown in Figures 3 and 4. Protein solubility showed the best peak at pH4 indicating a high protein solubility and a low isoelectric pH, which fell between pH4 and pH8. 18hrs of oven drying had the highest protein solubility compared to the rest of the oven‐dried flours. It was also observed that at pH10, the protein solubility was low for all the oven‐dried flours. Thus, pH10 was not a good pH for protein solubility for oven‐dried samples. For sun drying, flours had different peaks which indicated different isoelectric pH (pHi), implying many different types of proteins (more than 3 proteins). The best peak was observed at pH8 indicating a high protein solubility and a low isoelectric pH, which also fell between pH4 and pH8. The 5 days of sun‐dried flour had the highest protein solubility compared to the rest of the sun‐dried flours. It was also observed that at pH10, the protein solubility was high for the flour samples except for day 3. Thus, pH10 was a good pH for protein solubility for sun‐dried samples.

Generally, solubility reduces as pH increases, until it reaches the isoelectric point, followed by a progressive increase in solubility with further increase in pH. Similar observations have been presented earlier by Sathe et al. (1982) for Bran rice. It was observed that sun‐dried samples had a high protein solubility compared to the oven‐dried flour. The highest solubility of sun‐dried flour was found at a basic pH (pH10) while that of oven‐dried flour was at an acidic pH (pH4). The high solubility of oven‐dried flours in the acidic pH indicates that these flours may be useful in the formulation of acidic foods like cheese and yoghurt (Kinsella, 1979).

Kalaydzhiev et al. (2020) reported that most proteins have isoelectric points between pH 4.0 and 5.0. At the isoelectric point, there is no net charge on the protein, resulting in no repulsive interactions and the protein‐protein interactions, disfavoring solubility. At low pH, positive net charges of proteins are induced, while the proteins at high pH are conducted to the negative charge. Low protein solubility of oven‐dried flour may be due to protein denaturation since high temperatures cause irreversible denaturation and association followed by precipitation of high molecular weight compounds (Wolf & Tamura, 1969). Mild heat treatment on protein may not drastically reduce its solubility (Mulvihill & Donovan, 1987). Since protein solubility largely affects other functional properties such as emulsification, foaming, and gelation (Kinsella, 1976), the high solubility of the proteins for sun‐dried flour indicates that they could have promising food applications in the baking industry.

## 4 CONCLUSION

The results of the analyses of the functional properties of defatted *Annona muricata* seeds indicate its flours have technological aptitudes to be incorporated as an ingredient during feed or food formulation. Equally, the high degree of oil unsaturation (76% UFA and 31.82% PUFA), the low oil quality indices, combined with acceptable atherogenic index (less than 1) and good PUFA/SFA (> 0.8) as prescribed by legislation indicates that the oil may be healthy. Dehydration technique of postharvest storage of seeds (oven and sun‐drying) indicates that oven and traditional sun‐drying moderately affect the oil quality; sun‐drying remaining the most severe treatment for oil stability, despite the better functional properties (higher AOC, WAC, SI, and EA) of its derived defatted flours. Oven drying at 5℃ is recommended while sun‐drying of the seed for more than 4 days should therefore be prohibited. We, therefore, recommend that manufacturers of animal feeds should explore the potential use of soursop seeds for animal and human consumption.

## CONFLICT OF INTEREST

The authors have no conflict of interest to declare.

## AUTHOR CONTRIBUTION

**Fabrice TONFACK DJIKENG:** Data curation (equal); Visualization (equal). **Bernard Tiencheu:** Conceptualization (equal); Methodology (equal); Resources (equal); Supervision (equal); Writing‐original draft (equal). **Noel TENYANG:** Data curation (equal); Formal analysis (equal); Funding acquisition (equal); Methodology (equal). **Achidi Ufuan Aduni:** Supervision (equal); Visualization (equal); Writing‐review & editing (equal). **Tiepma Tiepma Ngongang:** Conceptualization (equal); Data curation (equal); Funding acquisition (equal); Software (equal). **Tatsinkou Fossi:** Conceptualization (supporting); Data curation (equal); Resources (supporting).
